# Oncogenic role of mortalin contributes to ovarian tumorigenesis by activating the MAPK–ERK pathway

**DOI:** 10.1111/jcmm.12905

**Published:** 2016-07-04

**Authors:** Yingying Hu, Ling Yang, Yujie Yang, Yanyan Han, Yongbo Wang, Wen Liu, Ji Zuo

**Affiliations:** ^1^ Department of Cellular and Genetic MedicineSchool of Basic Medical SciencesFudan UniversityShanghaiChina; ^2^ Obstetrics and Gynecology Hospital of Fudan UniversityShanghaiChina

**Keywords:** mortalin, ovarian cancer development and progression, MAPK–ERK

## Abstract

Mortalin is frequently overexpressed in human malignancies. Previous studies have suggested that mortalin contributes to ovarian cancer development and progression, but further investigation is warranted. The aim of this study is to elucidate the mechanism of mortalin in ovarian cancer development and progression. In this study, lentivirus‐delivered mortalin short hairpin RNA (shRNA) was used to knockdown mortalin expression in A2780 and A2780/cis ovarian cancer cell lines, and lentiviral mortalin‐pLVX‐AcGFP was used to generate mortalin‐overexpressing cell lines. The results demonstrated that decreased mortalin expression reduced ovarian cancer cell proliferation, colony formation, migration and invasion by Cell Counting Kit‐8 assay, colony formation assay, wounding healing assay and Transwell cell invasion assay, respectively. Flow cytometry results suggested that mortalin promotes the G1 transition, leading to faster restoration of a normal cell‐cycle distribution. Cell‐cycle proteins, including C‐myc and Cyclin‐D1, significantly increased, and Cyclin‐B1 remarkably decreased upon mortalin down‐regulation. Western blot analysis showed that mortalin knockdown significantly decreased p‐c‐Raf and phospho‐extracellular–regulated protein kinases (p‐ERK1/2) pathways but not the Jun N‐terminal kinase pathway, whereas mortalin overexpression had the opposite effect. Taken together, these results indicate that mortalin is an oncogenic factor, and mitogen‐activated protein kinase‐ERK signalling pathway activation by mortalin may contribute to ovarian cancer development and progression.

## Introduction

Ovarian cancer is the most lethal gynaecologic malignancy 1. The high mortality rate associated with ovarian cancer is observed because a high percentage of ovarian cancer patients are not diagnosed until an advanced stage 2. Tumour progression is a multi‐step process that advances cancer to a more malignant and aggressive phenotype 3. A high‐grade tumour represents a more advanced progression, in which the cancer cells possess higher proliferative and invasiveness capacities 4. Although significant advances have been made in ovarian cancer treatment, the survival rate is still poor and the overall cure rate remains low 5. Neoplasm recurrence and metastasis are considered the major reasons for poor clinical therapeutic and cancer deaths 6. Ovarian tumour grades are categorized in accordance with the International Federation of Gynecology and Obstetrics (FIGO) system, such that high‐grade tumours exhibit characteristics of faster cell growth, poor prognosis and drug resistance compared with low‐grade tumours 7, 8. Therefore, studying the mechanism of tumour proliferation and metastasis will provide further insights into ovarian cancer development and progression.

Mortalin, a molecular chaperone of HSP70 family, also known as glucose‐regulated protein 75 (Grp75), peptide‐binding protein 74 (PBP74) and mitochondrial heat shock protein 70 (mthsp70), is an essential protein that performs various functions related to proliferation, stress responses 9, mitochondrial biogenesis 10 and differentiation 11. Mortalin enrichment has been reported in several cancers, including leukaemia 12, brain cancer 13, colorectal adenocarcinoma 14 and hepatocellular carcinoma 15. Mortalin overexpression in colorectal adenocarcinomas was associated with malignant transformation and poor patient survival 16. At the same time increased mortalin expression in liver cancer was correlated with metastasis and early tumour recurrence 16. Furthermore, increased serum mortalin levels correlates with rapid disease progression and a risk factor in colorectal cancer patients 16. In addition, mortalin overexpression was sufficient to increase breast cancer cell malignancy 15. Ovarian cancer tissue microarray data has shown that mortalin was more highly expressed in advanced stages compared with lower stages of ovarian carcinomas and normal ovarian tissues 17. Mortalin up‐regulation and its association with increased tumour malignancy has been attributed to its ability to bind cytoplasmic p53 18. And mortalin can also activate AKT (also known as protein kinase B) in PC12 cells, which may be phosphoinositide 3‐kinase (PI3K) independent and associated with Raf/MEK/extracellular‐regulated protein kinases (ERK) signalling, and mortalin overexpression inhibited the Bax (a member of B‐cell lymphoma‐2) conformational change through the Raf/MEK/ERK signal pathway 19.

Because mortalin overexpression has been reported to contribute to tumorigenesis, we investigated its possible role and the underlying molecular mechanisms in ovarian cancer development and progression. These findings provide further insight for the oncogenic role of mortalin in mediating ovarian cancer tumorigenesis and raise the possibility that blocking mortalin expression may provide a new treatment approach for human ovarian cancer.

## Materials and methods

### Antibodies

Anti‐mortalin (#3593), anti‐p‐Erk1/2 (#4370), anti‐p‐Jun N‐terminal kinase (JNK; #4668), anti‐JNK (#9252), anti‐c‐Raf (#9421), anti‐p‐c‐Raf (#9422), anti‐Poly ADP ribose polymerase (PARP) (#9542), anti‐Cyclin‐D1 (#2978) and anti‐Cyclin‐B1 (#12231) were purchased from Cell Signaling Technology (Beverly, MA, USA). Anti‐C‐myc (sc‐764) was purchased from Santa Cruz Biotechnology (Santa Cruz, CA, USA). Anti‐β‐actin was purchased from Sigma‐Aldrich (St. Louis, MO, USA). Anti‐Erk1/2 (KC‐5E01) was purchased from Kang‐Chen Bio‐Tech, Inc. (Shanghai, China). Goat antimouse secondary antibodies (115‐035‐003) and goat anti‐rabbit secondary antibodies (111‐035‐003) were obtained from Jackson ImmunoResearch Laboratories, (West Grove, PA, USA).

### Cell culture

ES‐2, SKOV‐3, PM‐8910, HO‐8910, COC1, CAOV‐3, OV‐90, A2780 and A2780/cis cells were grown in DMEM supplemented with 10% foetal bovine serum (FBS; Biowest, Kansas, MO, USA) in a 5% CO_2_ humidified atmosphere at 37°C.

### Generation of mortalin‐expressing or shRNA lentiviruses

Mortalin‐overexpressing lentiviruses (mortalin‐pLVX‐AcGFP) and mortalin knockdown lentiviruses were generated in the pLVX‐AcGFP and PLKO.1 vectors, respectively, which are self‐inactivating and replication‐incompetent lentiviral green fluorescent protein‐expressing vectors, by inserting the mortalin PCR fragment and shRNA 17 into the BamHI/EcoRI sites of the vectors. Empty pLVX‐AcGFP and PLKO.1 vectors were used as controls. Mortalin overexpression primers were mortalin‐F: 5′‐ATCGCTCGAGGCCACCATGATAAGTGCCAGCCGAGCTGCA‐3′; mortalin‐R: 5′‐ATCGGGATCCTTACTGTTTTTCCTCCTTTTGATCT‐3′. Before transfection, 293T cells were seeded in a 10‐cm culture dish in DMEM medium supplemented with 10% FBS at a confluence of 40–50%. HilyMax (Dojindo, Kumamoto, Japan) was used to transfect the constructs following the manufacturer's instructions. The supernatant was collected 48 and 72 hrs post‐transfection and used for further infections.

### Whole‐cell protein extraction and Western blot analysis

Cells were lysed in RIPA buffer (150 mM NaCl, 1% NP‐40, 0.5% Doc, 0.1% SDS and 50 mM Tris/HCl, pH 8.0) supplemented with 100 μg/ml phenylmethylsulfonyl fluoride (PMSF). Protein concentrations were determined by Bradford assay (Thermo Pierce^®^ BCA Protein Assay Kit 23227; Thermo Fisher Scientific, Waltham, MA, USA). Proteins were separated on 10% SDS polyacrylamide gels and transferred onto protein‐blotted polyvinylidene membrane (GS0914; Millipore, Billerica, MA, USA). Membranes were blocked with 5% (wt/vol) non‐fat dry milk in TBS‐T buffer [20 mM Tris/HCl (pH 7.6), 137 mM NaCl and 0.05% Tween‐20] and incubated overnight at 4°C with relevant primary antibodies followed by washing and incubation with the appropriate horseradish‐peroxidase‐conjugated secondary antibodies. Finally, membranes were detected with Immobilon Western chemiluminescent HRP substrate (Bio‐Rad, Hercules, CA, USA), and images were captured with the Gel Doc XR System (Bio‐Rad). And the relative expression level of proteins was quantified by using Quantity One 1‐D analysis software (Bio‐Rad).

### RNA isolation and real‐time polymerase chain reaction

Total RNA was isolated from cells using Trizol reagent (Life Technologies, Carlsbad, CA, USA) according to the manufacturer's instructions. After quantification and determination of the quality of total RNA, first‐strand cDNA was synthesized with a RevertAid first‐strand cDNA synthesis kit (Thermo Fisher Scientific, Waltham, MA, USA). The mRNA level was determined by real‐time PCR (polymerase chain reaction) using SYBR Premix Ex Taq II (TaKaRa, Tokyo, Japan). PCR amplification cycles were programmed at 95°C for 30 sec., followed by 40 cycles of 95°C for 30 sec., 60°C for 30 sec. and 72°C for 40 sec. GAPDH was used as an endogenous control. Relative expression of genes was calculated and expressed using the 2^−ΔΔCt^ method. The primers used for real‐time PCR were as follows: GAPDH‐F, 5′‐TTGCCATCAATGACCCCTTCA‐3′; GAPDH‐R, 5′‐GAPDH‐RCGCCCCACTTGATTTTGGA‐3′; mortalin‐F: 5′‐AGCTGGAATGGCCTTAGTCAT‐3′ and mortalin‐R: 5′‐CAGGAGTTGGTAGTACCCAAATC‐3′.

### Cell viability assay

Cells were seeded at a density of 5 × 10^4^ cells/ml in 96‐well plates for 24 hrs. The cells were incubated with Cell Counting Kit (CCK‐8; CK04; Dojindo) for 3 hrs at 37°C. The optical density was determined at 450 nm using a Multiskan MK3 microplate reader (Thermo Fisher Scientific, Waltham, MA, USA).

### Colony formation assay

Cells were seeded in 60‐mm dishes at 250, 500 or 1000 individual cells per dish. Cells were maintained with regular changes in medium until colonies appeared. Colonies were fixed in methanol, stained with 10% Giemsa solution, photographed and counted.

### Wound healing assay

Cells were seeded in a six‐well plate until they reached full confluence in a monolayer. The medium in each well was then replaced with fresh medium containing Mitomycin C (10 μg/ml; Sigma‐Aldrich) to exclude the factor of increased cell growth and incubated overnight at 37°C. A single wound was created in the middle of each well using a micro‐pipette tip. The plate was incubated at 37°C and 5% CO_2_. Images of wound closure were acquired at different time courses. The relative migration rate was expressed as the relative width of the wounds/time.

### Transwell invasion assay

A total of 1 × 10^5^ cells were seeded on 8‐μm‐pore polycarbonate‐membrane Boyden chambers (Costar, Cambridge, MA, USA) coated with Matrigel (BD Biosciences, Bedford, MA, USA). A total of 700‐μl DMEM supplemented with a higher concentration of FBS was added to the lower chamber. After 24 hrs, cells on the top surface of the insert were removed by wiping with a cotton swab. Cells that had migrated to the bottom surface of the insert were fixed in methanol for 20 min., stained in crystal violet for 15 min., rinsed in PBS and examined microscopically (200×). The results represent the average number of cells per high‐power field (HPF) among 20 HPFs examined (*n* = 5).

### Cell‐cycle analysis by flow cytometry

Cells were harvested and fixed in 70% ethanol overnight and washed twice in cold PBS. The cells were then washed once with PBS and stained with 50 μg/ml propidium iodide at room temperature for 30 min. Cells were examined using a flow cytometer (BD Accuri C6; BD Biosciences, Bedford, MA, USA), and FlowJo software (FLOWJO, Ashland, OR, USA) was used for flow cytometry (FCM) analysis.

### Statistical analysis

Data were expressed as the mean ± S.E.M. in at least three independent experiments. Statistical significance was determined using Student's *t*‐test or an anova. *P* < 0.05 was considered statistically significant.

## Results

### Mortalin is up‐regulated in ovarian cancer cell lines and it promotes ovarian cancer cell growth

**Figure 1:**
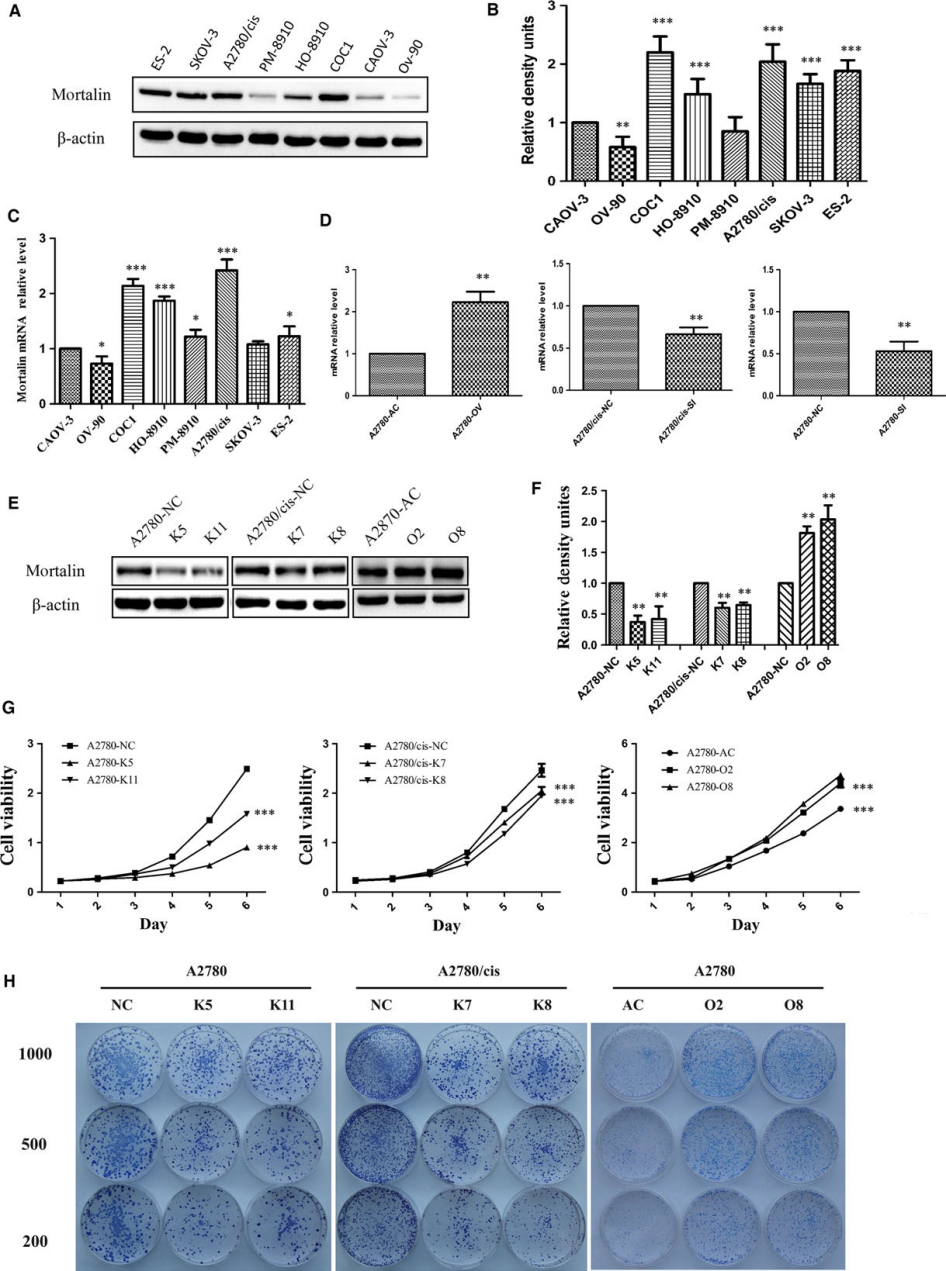
Mortalin is up‐regulated in ovarian cancer cell lines and promotes ovarian cancer cell growth. (**A**) Western blot analysis using anti‐mortalin antibody to evaluate mortalin (75 kD) expression in ovarian cancer cell lines. (**B**) Quantitative results from Western blots. β‐actin was used as internal control. (**C**) Mortalin mRNA expression in ovarian cancer cells was analysed using Quantitative RT‐PCR. (**D**) To confirm the transfection efficiency, mortalin mRNA expression in different transfected cells was analysed through Quantitative RT‐PCR analyses. (**E**) Western blot showed mortalin protein expression in different groups. Reduced mortalin expression in stable knockdown clones generated by shRNA (K5 and K11 of A2780, K7 and K8 of A2780/cis). Scrambled cells (A2780‐NC and A2780/cis‐NC) were the non‐specific shRNA control groups. Stable mortalin‐expressing clones (O2 and O8) were established in A2780 cells, Control cells (A2780‐AC) were transfected by empty vectors. (**F**) Quantitative results from Western blots. β‐actin was used as a loading control. (**G**) CCK‐8 cell proliferation assay demonstrated that mortalin overexpression in A2780 cells induced significantly higher growth rates compared with their vector controls. In contrast, mortalin depletion in A2780 and A2780/cis cells remarkably decreased cell proliferation compared with their scrambled controls. (**H**) Colony formation assay showed that mortalin‐overexpressing cells formed much larger clones. *P < 0.05, **P < 0.01, ***P < 0.001.

To demonstrate the oncogenic role of mortalin in ovarian cancer cell lines, we performed Western blot and real‐time PCR analyses to measure mortalin expression in eight human ovarian cancer cell lines (ES‐2, SKOV‐3, A2780/cis, PM‐8910, HO‐8910, COC1, CAOV‐3 and OV‐90). We already know from previous study, mortalin is up‐regulated in CAOV‐3 cells 15. Our Western blot result (Fig. 1A and B) showed that mortalin expression is higher or similar in other seven cell lines compare with the mortalin expression in CAOV‐3 cells. And Figure 1C showed that mortalin mRNA was up‐regulated in ES‐2, SKOV‐3, A2780/cis, PM‐8910, HO‐8910 and COC1 cells compared with CAOV‐3 and OV‐90. Compared with a low‐grade ovarian cancer cell line (OV‐90 and CAOV‐3), metastatic tumour cells (COC1) and cisplatin‐resistant ovarian cancer cells (A2780/cis) have elevated mortalin expression.

We chose cisplatin‐sensitive A2780 and cisplatin‐resistant A2780/cis ovarian cancer cell lines for further studies. A2780 cells were infected with lentiviral mortalin to construct mortalin high‐expression cell lines (A2780‐OV), and the pLVX‐AcGFP lentiviral vector was used to construct the control cells (A2780‐AV). At the same time, shRNA was employed to silence mortalin expression in A2780 and A2780/cis cells (A2780‐SI and A2780/cis‐SI), and A2780‐NC and A2780/cis‐NC cells stably transfected with the negative shRNA were used as respective control groups.

Quantitative real‐time PCR assay was used to demonstrate the transfection efficiency. Results (Fig. 1D) showed that mortalin mRNA expression in A2780‐OV cells were higher compared with A2780‐AC cells, and mortalin mRNA expression in A2780‐SI and A2780/cis‐SI cells was lower compared with A2780‐NC and A2780/cis‐NC cells, respectively. Western blot analysis was used to choose two groups of the most efficient cell lines: K5 and K11 of A2780 stably expressing anti‐mortalin shRNA, K7 and K8 of A2780/cis stably expressing anti‐mortalin shRNA, O2 and O8 of A2780 cells stably expressing mortalin (Fig. 1E and F).

We investigated the effect of mortalin on cell proliferation of ovarian cancer cells through CCK‐8 cell proliferation assay (Fig. 1F) and colony formation assay (Fig. 1G). Cells in A2780‐O2 and A2780‐O8 groups had significantly present higher growth rates and formed much larger clones than cells in A2780‐AC group. In contrast, depletion of endogenous mortalin in A2780 and A2780/cis cells remarkably slowed down cell proliferation compared with the scrambled controls. These results demonstrated that mortalin is essential for ovarian cancer cell growth.

### Altered mortalin levels regulate ovarian cancer cell migration and invasion

**Figure 2:**
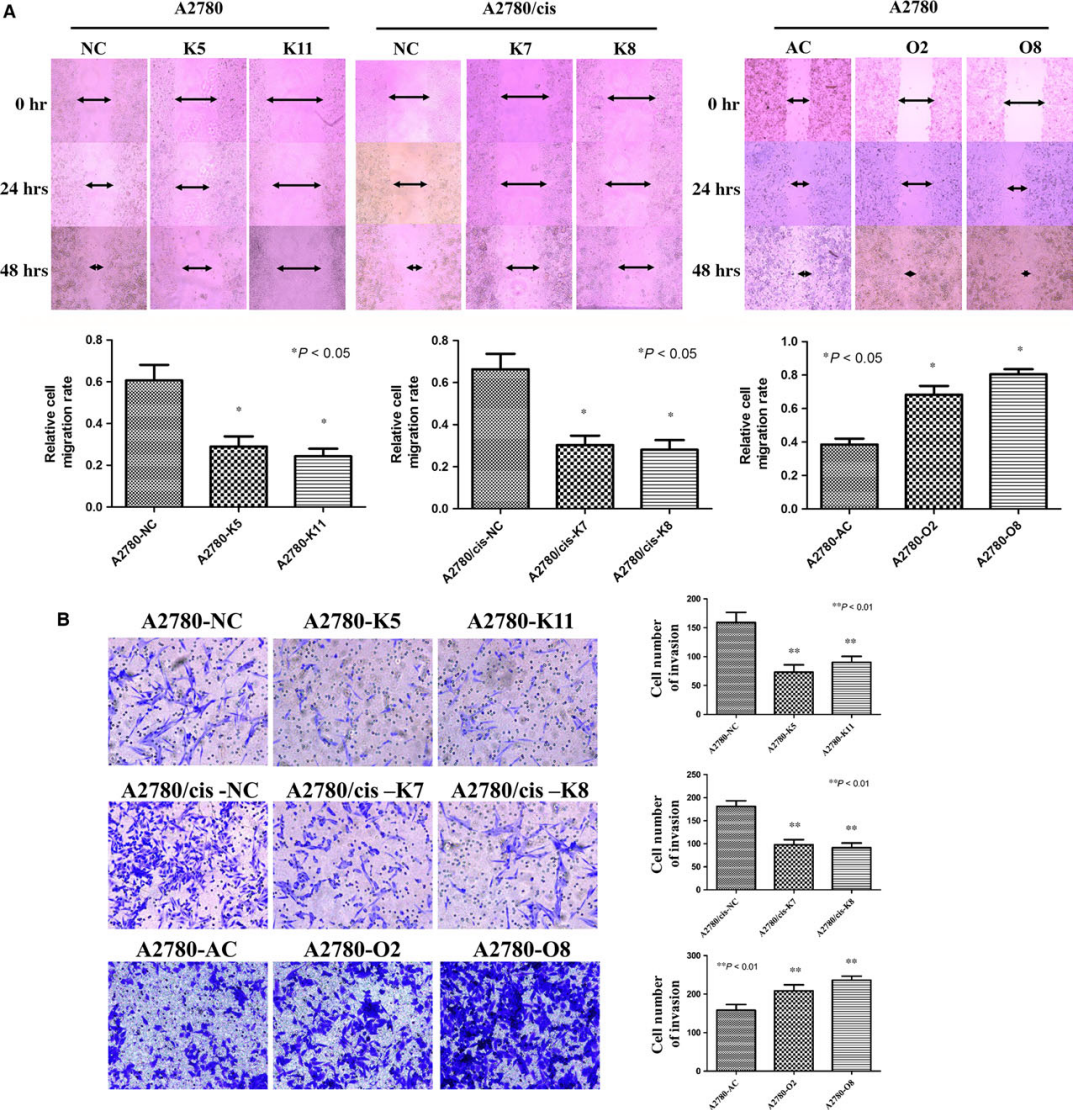
Mortalin promotes ovarian cancer cell migration and invasion. (**A**) Wound healing assay showed that the wound closure rate was significantly reduced in A2780‐K5, A2780‐K11, A2780/cis‐K7 and A2780/cis‐K8 cells compared with A2780‐NC and A2780/cis‐NC cells, respectively, in the time course of 24 and 48 hrs. At the same time, the wound closure rate was higher in A2780‐O2 and A2780‐O8 cells compared with A2780‐AC cells in the time course of 24 and 36 hrs. The arrows indicate the wound width, and the relative cell migration rate is expressed as relative width of the wounds/time. (**B**) Transwell invasion assay demonstrated that mortalin stable knockdown reduced cell invasiveness compared with the scrambled controls. Mortalin‐overexpressing clones invaded faster through the matrigel compared with the vector control. Three views were randomly picked in each insert, and the numbers of invaded cell was counted. *P < 0.05, **P < 0.01.

Migration and invasion are fatal steps in cancer progression, with death from metastases accounting for 90% of all human cancer mortalities 20. Therefore, it is important to identify therapeutic targets to prevent the spread of cancer cells. Here, we examined whether mortalin plays a role in accelerating ovarian cancer cell migration and invasion. We first performed wound healing assay to examine whether mortalin expression promotes cell migration. The results showed (Fig. 2A) that forced expression (O2 and O8) of mortalin in A2780 cells induced a faster wound closure rate than in the vector control (A2780/NC). In contrast, mortalin down‐regulation in A2780 (K5 and K11) and A2780/cis (K7 and K8) cells significantly reduced the cell migration rate (Fig. 2A). Furthermore, transwell invasion assay demonstrated (Fig. 2B) that the number of cells that penetrated through Matrigel was higher in mortalin‐overexpressing clones (A2780‐O2 and A2780‐O8) compared with the vector control (A2780‐AC). In contrast, fewer cells in stable mortalin knockdown clones (A2780‐K5, A2780‐K11, A2780/cis‐K7 and A2780/cis‐K8) passed through the filter compared with the respective scrambled controls (Fig. 2B). These data suggest that increased mortalin expression promotes ovarian cancer cell migration and invasion.

### Mortalin promotes ovarian cancer cell‐cycle progression through Cyclin‐D1

**Figure 3:**
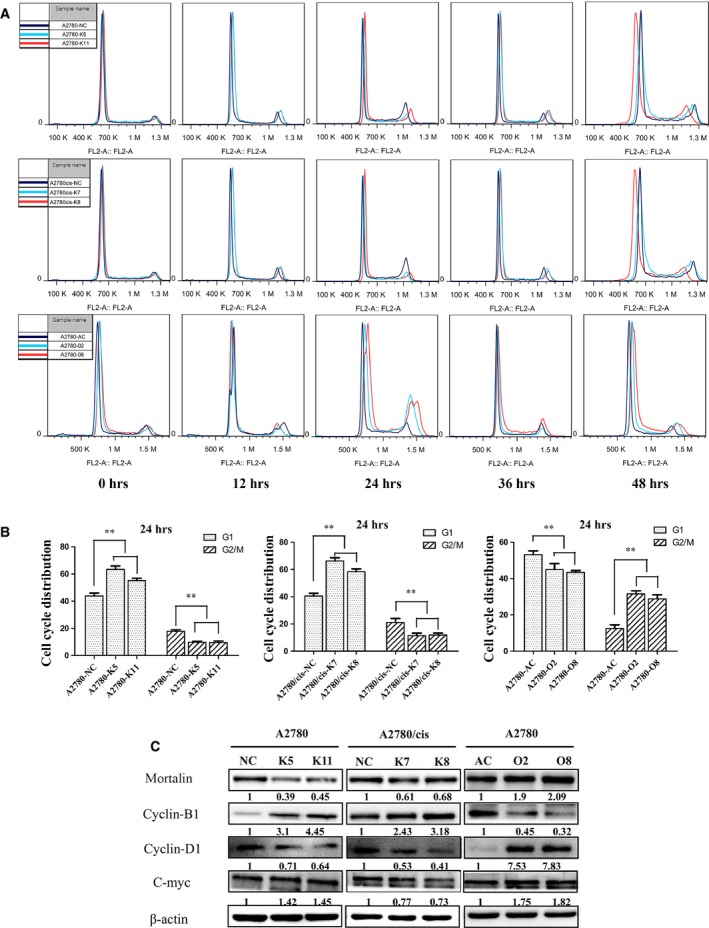
Mortalin promotes ovarian cancer cell proliferation and accelerates cell‐cycle progression. (**A**) Cells were deprived of serum for 72 hrs and were then released from G1 arrest by adding serum, then subjected to FCM analysis at the indicated time points (0, 12, 24, 36 and 48 hrs). (**B**) Statistical analysis of the G1 and G2/M phase at 24 hrs (*n* = 3), **P < 0.01. (**C**) Western blot analysis showed that Cyclin‐B1, Cyclin‐D1 and C‐myc protein levels changed with varied mortalin expression. β‐actin was used as a loading control. The numerical value under each panel represents the relative expression of mortalin, Cyclin‐B1, Cyclin‐D1 and C‐myc to their vector controls.

To better determine the impact of mortalin overexpression on cell growth, we performed cell‐cycle analysis using FCM. Cells were synchronized in the G1 phase by serum starving cells for 72 hrs. After the addition of serum, cells with lower mortalin expression sequentially proceeded through S and G2/M phase and approached a normal cell‐cycle phase distribution by 48 hrs. In contrast, cells‐expressing higher mortalin levels progressed through the G1 phase to the G2/M phase by 24 hrs. These data suggest that mortalin promotes the G1 transition, leading to a faster restoration of the normal cell‐cycle distribution (Fig. 3A and B). Cyclin‐B1, Cyclin‐D1 and C‐myc are three crucial regulators of cell proliferation 21, 22. Western blot analyses (Fig. 3C) demonstrated that both Cyclin‐D1 and C‐myc expression increased in mortalin‐overexpressing ovarian cancer cells (A2780‐O2 and A2780‐O8) compared with A2780‐AC, whereas Cyclin‐B1 levels decreased. In contrast, Cyclin‐D1 and C‐myc expression decreased in mortalin knockdown groups (A2780‐K5, A2780‐K11, A2780/cis‐K7 and A2780/cis‐K8) compared with their respective control groups (A2780‐NC or A2780/cis‐NC), and Cyclin‐B1 levels increased in those mortalin knockdown cells. These results demonstrate that mortalin expression reduces Cyclin‐B1 and up‐regulates Cyclin‐D1 and C‐myc to promote ovarian cancer cell growth.

### Mortalin expression levels affect MAPK–ERK pathway activation in ovarian cancer cells

**Figure 4:**
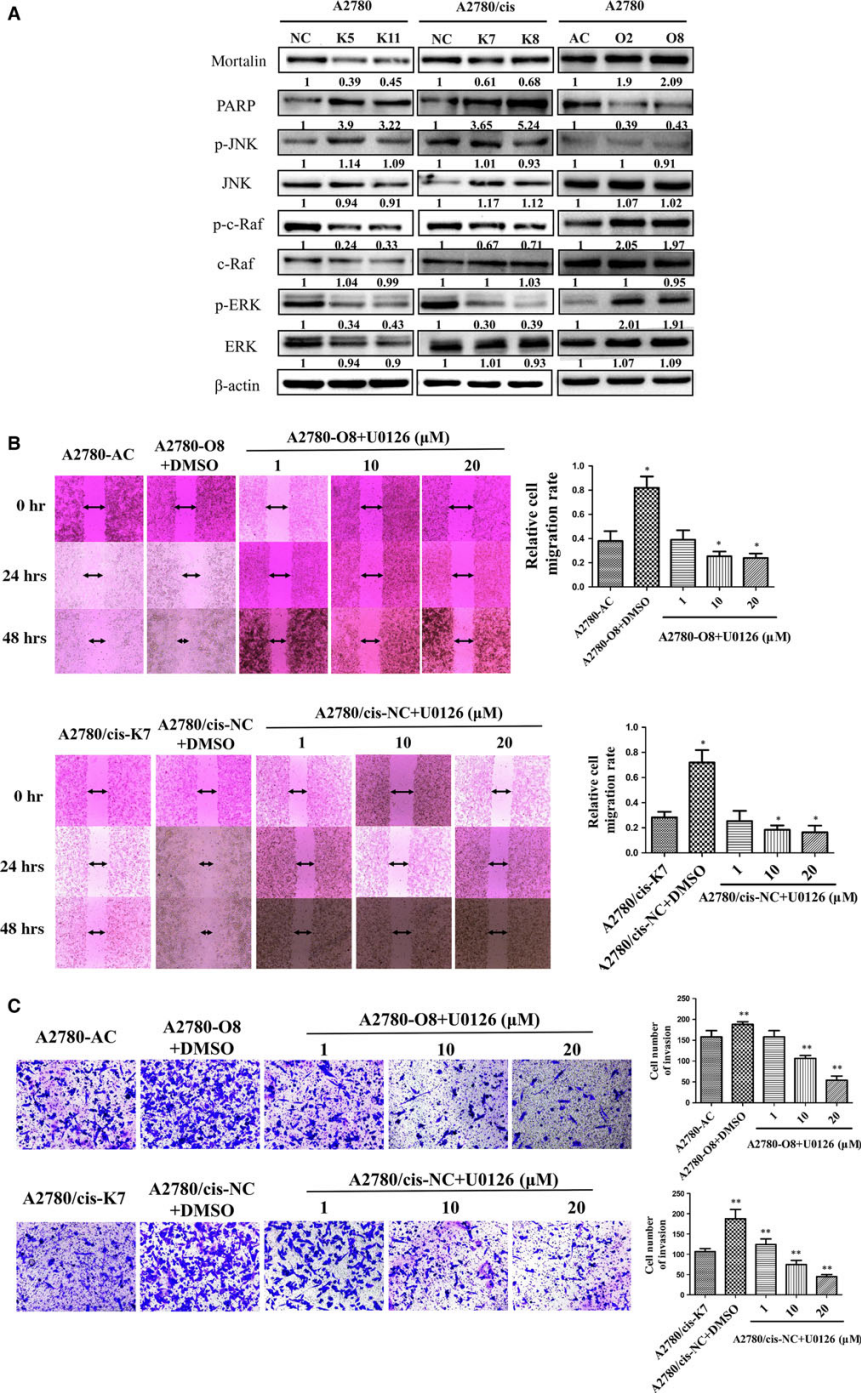
Mortalin expression levels affect MAPK–ERK pathway activation in ovarian cancer cells. (**A**) Changes in signal transduction under mortalin overexpression or down‐regulation in A2780 and A2780/cis cells detected by Western Blot. The numerical value under each panel represents the relative expression of targeted proteins to their vector controls. (**B**) U0126, a specific inhibitor of MEK, was used to the A2780‐O8 and A2780/cis‐NC cells for 2 hrs before the wound healing assay performed. U0126 pre‐treated A2780‐O8 and A2780/cis‐NC cells exhibited lower wound closure rate than their DMSO pre‐treated cells (*P < 0.05). (**C**) U0126 was used to the A2780‐O8 and A2780/cis‐NC cells for 2 hrs before the transwell invasion assay performed. U0126 pre‐treated A2780‐O8 and A2780/cis‐NC cells invaded lower through the matrigel compared with their DMSO pre‐treated cells, respectively (**P < 0.01).

Intracellular signal transduction pathways have often shown crosstalk on multiple levels. To explore the major molecular events in ovarian cancer cells induced by varied mortalin expression, we chose to examine several antibodies for cell signalling pathways in the altered mortalin cell lines. As shown in Figure 4A, the p‐ERK1/2 and p‐c‐Raf levels increased in mortalin overexpression (A2780‐O2 and A2780‐O8) cells compared with A2780‐AC cells, and the p‐ERK1/2 and p‐c‐Raf levels decreased in mortalin knockdown cells (A2780‐K5, A2780‐K11, A2780/cis‐K7 and A2780/cis‐K8) compared with their control groups. However, the expression of ERK1/2 and c‐Raf did not change significantly in all groups. At the same time, PARP increased in mortalin knockdown cells and decreased in mortalin overexpression cells compared with their respective control cells. We did not observe any significant changes in JNK or p‐JNK expression. These data suggest that mortalin may contribute to ovarian cancer development through the mitogen‐activated protein kinase (MAPK)–ERK signal pathway, but not the JNK signal pathway.

To make sure the relationship between mortalin contribution to ovarian cancer development and the MAPK–ERK signal pathway, U0126, a specific inhibitor of MEK, was used to further investigation. We added different concentration of U0126 (1, 10 and 20 μM) to the A2780‐O8 and A2780/cis‐NC cells for 2 hrs, then the wound healing assay and transwell invasion assay were performed. Dimethyl sulfoxide (DMSO) was used as control. Results (Fig. 4B and C) showed that both the cell migration rate and cell number of invasion decreased in U0126 pre‐treated A2780‐O8 and A2780/cis‐NC cells compared with their respective DMSO pre‐treated control cells. All these results were consistent with the decreased p‐ERK expression. Western blot was used to measure the expression of mortalin in U0126 and DMSO pre‐treated cells. Results (Figs S1A, B and S2A, B) showed U0126 decreased p‐ERK1/2 in a concentration‐depended manner. So we concluded that mortalin can active the MAPK–ERK pathway, and then influence the development of ovarian cancer.

## Discussion

Mortalin, a mitochondrial chaperone, plays an important role in maintaining mitochondrial function 23. Mortalin overexpression has been detected in various tumour types 12, 13, it plays a role in carcinogenesis 15 and confers chemotherapeutic drug resistance. Mortalin was particularly up‐regulated in high‐grade ovarian cancer tissues 17. A mechanism of mortalin expression and its effect on ovarian cancer development and progression remains unknown.

Here, we present evidence that mortalin was frequently up‐regulated in eight ovarian cancer cell lines using real‐time PCR and Western blot analyses. According to the FIGO grading classification, high‐grade ovarian tumour cells grow faster and are highly metastatic 7. In addition, the prognosis of high‐grade ovarian tumours is poor and is often associated with a poor survival rate 24, 25. Our study demonstrated that both mortalin mRNA and protein levels were frequently up‐regulated in ovarian cancer cells, particularly in metastatic (COC1) and drug‐resistant cell lines (A2780/cis), suggesting that mortalin plays an important role in driving aggressive ovarian cancer phenotypes. To elucidate our hypothesis, we examined mortalin's oncogenic role in cell proliferation, anchorage‐independent growth, cell migration and invasion.

To determine the functional roles of mortalin in ovarian cancer, we generated mortalin‐overexpressing or knockdown ovarian cancer cell lines and performed a series of assays to examine the effect of mortalin expression on cancer cell proliferation and metastasis. Our study demonstrated that forced mortalin expression significantly enhanced cell proliferation, anchorage‐independent growth and cell migration/invasion. Conversely, reduced mortalin expression by shRNA impaired the above tumorigenic phenotypes. Taken together, our findings suggest that the oncogenic functions possessed by mortalin are closely correlated with high‐grade ovarian cancer.

Uncontrolled cell division is an indispensable event in tumour progression, and numerous molecules have been implicated in this process. Unlike normal cells that transiently express small amounts of cyclin proteins at specific points in the cell cycle, many tumours express abnormal levels of one or more cyclins 26. Previous studies have shown that the immune system recognizes some abnormally expressed cyclins as tumour antigens, such as c‐myc, Cyclin‐B1 and Cyclin‐D1 21, 27, 28. Furthermore, oncogene activation, such as Cyclin‐D1 and C‐myc, can enhance anchorage‐independent growth of mouse mammary epithelial cells and tumour growth in severe combined immunodeficiency mice 21. These data suggest that cyclin regulation contributes to more aggressive tumour features, including metastasis. Thus, we speculate that mortalin expression increases cell proliferation and anchorage‐independent growth by up‐regulating these genes. In support of our hypothesis, FCM showed that mortalin promotes the G1 transition, leading to faster restoration of the normal cell‐cycle distribution. Cyclin‐B1 expression was down‐regulated, whereas both Cyclin‐D1 and C‐myc were up‐regulated upon mortalin overexpression. In contrast, mortalin knockdown ovarian cancer cells showed increased Cyclin‐B1 levels and decreased Cyclin‐D1 and C‐myc levels. Collectively, these results demonstrate that mortalin mediates tumour progression by acting as an oncogenic factor by activating downstream oncogenes.

Mortalin and other HSPs can activate cell survival pathways and inhibit apoptosis pathways 19, 29. Raf/MEK/ERK is an intracellular signalling pathway that promotes cell survival 30. Erk1/2 plays a critical role in preventing apoptosis and regulating cell survival 31, and c‐Raf has a MEK/ERK‐independent role that is often involved in cell‐cycle progression regulation 32. Thus, we speculated that increase mortalin in ovarian cancer is one genetic alteration that provides constitutive MAPK–ERK signalling activation and drives cell proliferation and tumour formation. Our previous study showed that there may be some relationship between mortalin contribution to ovarian cancer development and MAPK–ERK pathway 33. In support of this hypothesis, we also observed C‐myc and Cyclin‐D1 up‐regulation in mortalin‐overexpressing cells. This is a logical result of MAPK–ERK pathway activation, as both C‐myc and Cyclin‐D1 are oncogenic elements implicated in ovarian cancer development.

Previous studies demonstrated that the inhibition of mortalin expression suppresses A2780/cis cell proliferation and invasion and increases cisplatin‐induced apoptosis 17. Consistently, our data showed that mortalin knockdown increased PARP cleavage. Western blot analysis indicated that mortalin expression induced Raf1–MEK1/2–ERK1/2 cascade activation, but not c‐JNK pathway activation, as demonstrated by Raf1, MEK1/2 and ERK1/2 phosphorylation, demonstrating the role of mortalin in MAPK–ERK pathway regulation. Then the inhibitor of MAPK–ERK pathway, U0126, was used to confirm the results. After the p‐ERK1/2 expression decreased by U0126, the cell migration rate and cell number of invasion decreased at the same time. All the results consistent with the p‐ERK1/2 expression reduced by knockdown of mortalin. So we concluded that mortalin may contribute to ovarian cancer development and progression through MAPK–ERK pathway.

Consistently, MAPK–ERK activation leads to Cyclin‐B1, c‐myc and Cyclin‐D1 regulation, resulting in increased cell proliferation and accelerated G1 transition, suggesting that the ERK–myc–Cyclin D1 axis can be, at least partly, an oncogenic mechanism by which mortalin contributes to ovarian cancer development and progression. As intracellular signal transduction pathways often show crosstalk with others, whether mortalin directly or indirectly affects the MAPK–ERK pathway requires further research.

In conclusion, our findings suggest that mortalin plays an important role in ovarian cancer development and progression by promoting tumour growth and migration/invasion in ovarian cancer, and mortalin is involved in the modulation of cell‐cycle–related proteins and the MAPK–ERK signalling pathway. Ovarian cancer cell growth and survival can be inhibited by mortalin inactivation, suggesting the oncogenic role of mortalin and indicating a potential novel target for chemotherapy.

## Conflict of interest

The authors declare that there are no conflicts of interest.

## Supporting information


**Figure S1** Effects of MEK inhibitor (U0126) on the expression of A2780‐O8 cells. (**A**) Different concentration (0.1, 1, 10 and 20 μM) of U0126 and DMSO was administered 2 hrs and the phosphorylation level of ERK1/2 was measured by Western blot. β‐actin was used as a loading control. (**B**) Quantitative results from Western blots. β‐actin was used as internal control.

**Figure S2** Effects of MEK inhibitor (U0126) on the expression of A2780/cis‐NC cells. (**A**) Different concentration (0.1, 1, 10 and 20 μM) of U0126 and DMSO was administered 2 hrs and the phosphorylation level of ERK1/2 was measured by Western blot. β‐actin was used as a loading control. (**B**) Quantitative results from Western blots. β‐actin was used as internal control.
