# Supplementary figures and images for: Oncogenic role of mortalin contributes to ovarian tumorigenesis by activating the MAPK–ERK pathway
Source: J Cell Mol Med. 2016 Jul 4;20(11):2111–21. doi: 10.1111/jcmm.12905 (PMC5082394; doi:10.1111/jcmm.12905)

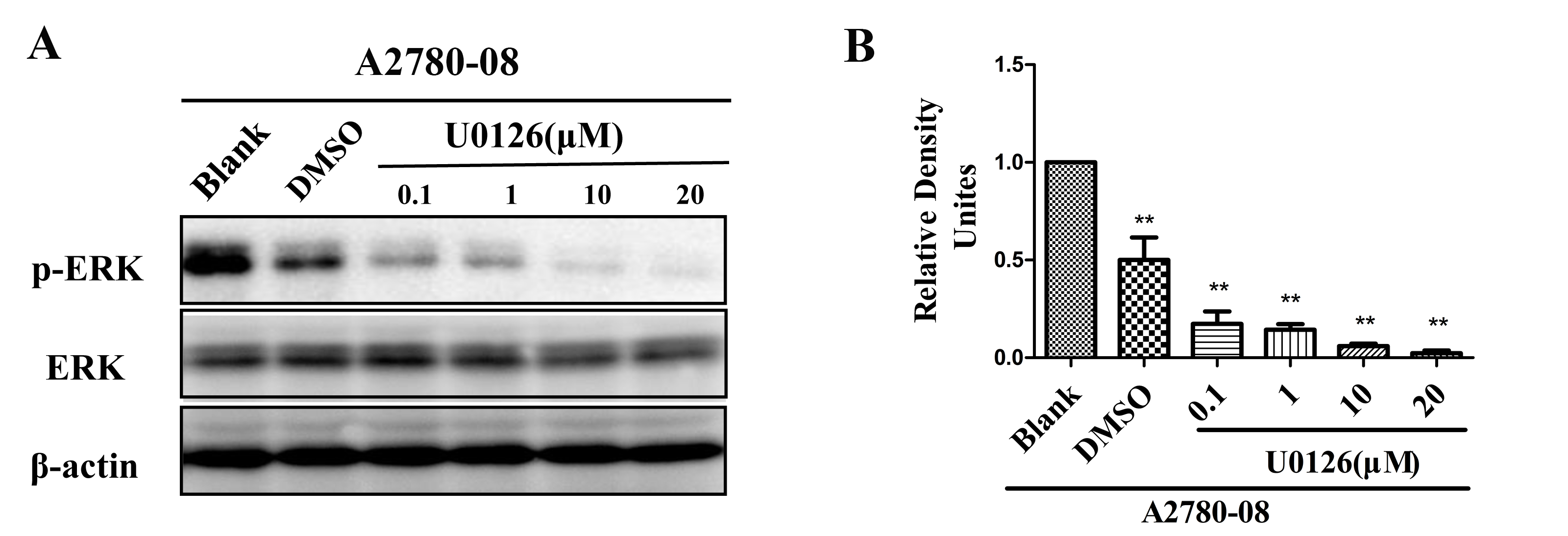

Supplement: Supplementary file 1 — Figure S1 Effects of MEK inhibitor (U0126) on the expression of A2780‐O8 cells. (A) Different concentration (0.1, 1, 10 and 20 μM) of U0126 and DMSO was administered 2 hrs and the phosphorylation level of ERK1/2 was measured by Western blot. β‐actin was used as a loading control. (B) Quantitative results from Western blots. β‐actin was used as internal control. [file JCMM-20-2111-s001.tif]

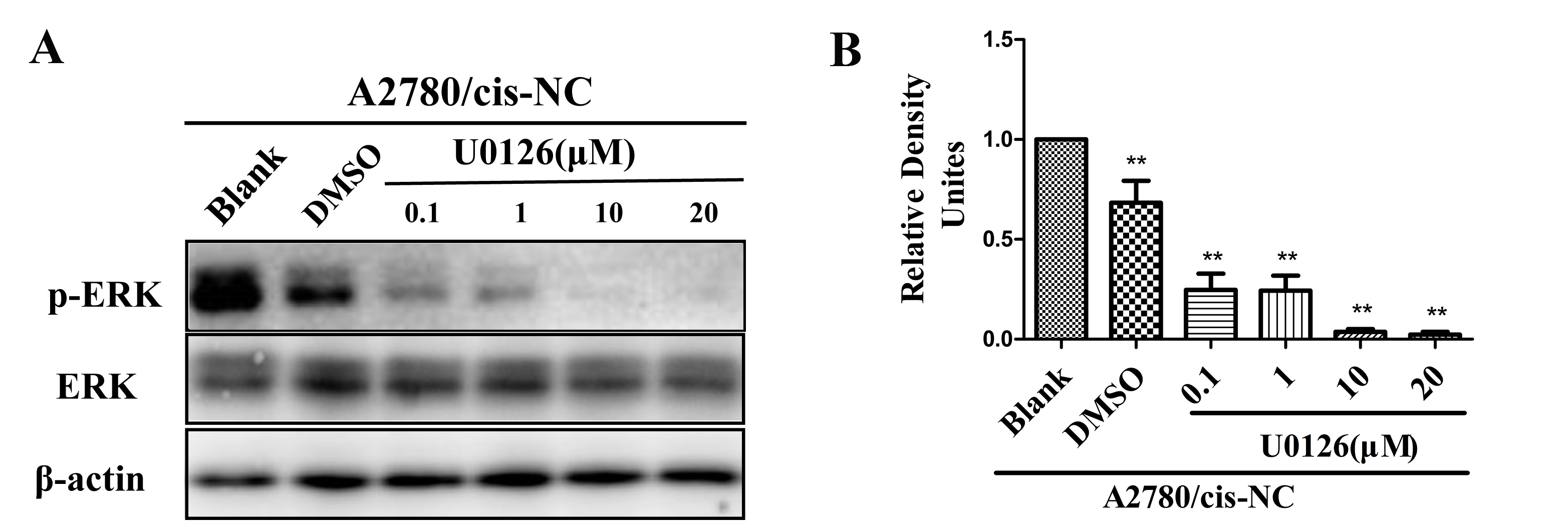

Supplement: Supplementary file 2 — Figure S2 Effects of MEK inhibitor (U0126) on the expression of A2780/cis‐NC cells. (A) Different concentration (0.1, 1, 10 and 20 μM) of U0126 and DMSO was administered 2 hrs and the phosphorylation level of ERK1/2 was measured by Western blot. β‐actin was used as a loading control. (B) Quantitative results from Western blots. β‐actin was used as internal control. [file JCMM-20-2111-s002.tif]
